# Moroccan City Festivals, Cultural Diplomacy and Urban Political Agency

**DOI:** 10.1007/s10767-020-09390-4

**Published:** 2020-10-12

**Authors:** Nick Dines

**Affiliations:** Department of Sociology and Social Research, Università degli Studi Milano Bicocca, Via Bicocca degli Arcimboldi 8, 20126 Milan, Italy

**Keywords:** Festivals, Cultural diplomacy, Urban agency, State rescaling, Morocco, Rabat, Marrakech

## Abstract

Over the last two decades, cultural festivals have been established and consolidated in cities across Morocco. Their proliferation has coincided with the reign of Mohammed VI, well known as an enthusiastic and extremely wealthy patron of the arts, and the concomitant state-controlled democratization of Moroccan politics and society. Drawing on two examples—the Marrakech International Film Festival and the Mawazine music festival in Rabat—this article interrogates the ways in which festivals and the urban scale combine to function as vehicles for cultural diplomacy. Contra the common tendency in recent policy debates that perceive the city (with or without its administration) as an active agent in translocal cultural relations, I argue for a more nuanced perspective that understands the urban festival as a diplomatic platform through which the cultural politics of the state are rescaled and where a range of actors contest ideas about the local, national and global trajectories of society and cultural life.

## Introduction

During the last two decades, numerous cultural festivals have been established and consolidated in cities across Morocco. Their proliferation has coincided with the reign of Mohammed VI and a concomitant process of democratization of Moroccan politics and society. The array of music, film, theatre and art festivals have showcased a mixture of Moroccan and international performers and cultural products, and have often been directly run by or received the approval and financial backing from the Royal Palace and its inner circle, the Makhzen. Drawing on local and international media coverage, festival publicity and secondary literature on Moroccan festivals and cultural policy, as well as personal insights gathered through ongoing research conducted in the field, this article interrogates the ways in which festivals function specifically as vehicles for cultural diplomacy. Focusing on two different cases—the Marrakech International Film Festival and the Mawazine music festival in Rabat—it also reflects more broadly on the diplomatic relevance of cities in a particular non-Western context.

In order to begin to comprehend how festivals and their urban settings are mobilized by different actors to establish translocal relations, it is important to first distinguish between *city diplomacy*, on the one hand, and *the role of cities in cultural diplomacy*, on the other. While there may be some overlap between these two domains, they are not the same thing. City diplomacy (Viltard 2008, 2010; Acuto 2013; Curtis and Acuto 2018) can be defined as ‘the institutions and processes by which cities or local governments in general, engage in relations with actors on the international political stage with the aim of representing themselves and their interests to one another’ (Van der Pluijm 2007, p.6). City diplomacy is seen to normally taking place in fora, such as transnational municipal networks, where representatives of cities come into contact with each other. Cultural diplomacy, meanwhile, is a semantically broad field that refers to any practice involving ‘purposeful cultural cooperation’ between sub-national, national and supranational entities (Ang et al. 2015, p. 366). Cultural diplomacy does not necessarily involve formal exchanges: on the contrary, it is often initiated independently by one party for the purposes of, say, self-promotion or cultural pedagogy. Moreover, initiatives can occur at home or abroad, and the intended audience is not, as a matter of course, a direct counterpart but can vary according to circumstances. Hence, a national government may embark on a cultural diplomatic offensive that targets a particular world region or a specific group within a state. For example, the former US diplomat, Cynthia Schneider believed that the principal mission of the US government’s cultural diplomacy during the early 2000s was to reach out to the Arab world generally and young people in particular in order to improve the country’s tattered reputation following the invasion of Iraq (Schneider 2006, p. 191). Similarly, the practice of cultural diplomacy at the urban scale does not target other cities alone but a host of different and not always clearly defined audiences.

The relationship between cultural diplomacy and cities raises a number of issues that have been at the centre of recent debates in urban studies and international relations, namely urban political agency and global urban governance (Amen et al. 2011; Oosterlynck et al. 2019). According to influential public thinkers such as Edward Glaeser (2011) and the late Benjamin Barber (2013), cities are better positioned than nation states to politically tackle global questions such as climate change and diversity management. City governments in recent years have assumed increasingly high profiles in international affairs, in part due to the commanding positions of certain cities in the global economy. This has been accompanied by a proliferation of inter-urban networks, such as the C40 Cities Climate Leadership Group and United Cities and Local Government (UCLG).

Critical scholars have challenged the ‘urban triumphalism’ of the likes of Barber and Glaeser for underplaying the deep inequalities that exist within and between the world’s cities (Bouteligier 2013) and have argued that many city networks and their value systems operate as foils for neoliberal development models and corporate interests (Bréville 2020), while others have disputed the common accompanying assumption that ‘nation-states and state-processed national economies [have become] unimportant to the world economy and world/global cities’ (Therborn 2011, p. 272). Taking their cue from the pioneering work of Neil Brenner (2004), urban researchers, including those working on Moroccan cities (Bogaert 2012, 2018a), have explored the formation of new state spaces at the urban scale which reflect the ‘reorientation of state spatial strategies from nationally redistributive modalities towards urban-centric, competitiveness-oriented forms of locational policy’ (Brenner 2009, p. 129). As a conducive site for new governance arrangements and capital accumulation, the city does not function as a separate domain but is one that is embedded in multilevel governance frameworks and shaped by global *and* national flows of capital. Furthermore, the urban scale is never pre-given but is ‘the outcome of power struggles in a given geohistorical setting’ (Bassens et al. 2019, p. 8), in particular the conflict-ridden shift from the post-war Keynesian settlement towards a neoliberal market economy that occurred in the West after the 1970s and which was subsequently imposed upon much of the Global South through World Bank and IMF-sponsored structural adjustment programmes (Ferguson 2006).

Alongside the rescaling and reterritorialization of state power, researchers have also highlighted the limits of conflating urban political agency with mayors and/or local administrations. Bassens et al. (2019) have argued for a more plural conceptualization of urban political agency that sees power as diffuse and comprising multiple constituents—state and non-state institutions, public-private coalitions, lobbyists, civil society, grassroots pressure, etc.—that try, with varying degrees of success, to influence local politics. Moreover, the city’s heralded capacity for autonomous action has been tempered by major global-scale events in recent years, from the 2008 financial crisis to the COVID-19 pandemic. As the sociologist Göran Therborn caustically observes, following the 2008 crisis ‘cities […] turned out to be policy *takers*, rather than freely navigating on global flows without territorial moorings’ (Therborn 2011, p. 275 emphasis added).

It therefore follows that the city should not be viewed as a unitary actor. Even though it may sometimes offer a tempting shorthand, we must beware of anthropomorphizing the city into an active subject with its own unified agency. In other words, ‘the city’ does not *do* cultural diplomacy. Rather, different actors deploy the city or operate through the urban scale to transmit cultural messages targeted at, or cultivate relations with, other groups, localities and nations.

This paper explores how cultural diplomacy vis-à-vis urban political agency and the rescaling of state power unfolds in a context outside the so-called global North and beyond the (less than clear-cut) boundaries of Western liberal democracy (Natter 2018). A focus on non-Western cities conventionally considered to possess marginal roles in the global economy and hierarchies of cultural value encourages us to interrogate the ‘horizontal’ character of translocal networks and consider how power relations within them intensify existing inequalities between ‘informational and ideational flows’ (Bouteligier 2013). We also need to take into account the fact that the strategic decisions taken to cultivate translocal relations may be geared as much to establishing ‘south-south’ connections as they are to pursuing Western urban or cultural repertoires. This is illustrated by the development of Kazakhstan’s new capital city Astana/Nur-Sultan. As Natalie Koch observes, while the Kazakh authorities and planners shared similar policy ideas to those in Barcelona or Berlin, insofar as they similarly sought to stake a place for the city in the global urban order, the city’s construction was legitimated ‘in a different fashion than the supposedly hegemonic discourses of free markets and political liberalism’ and its inspiration was drawn from other urban centres, notably the Turkish capital, Ankara (Koch 2013, p. 124). The motivations for why cities may engage in certain international relations and not others arise from a complex interplay of local, national and global interests. It is crucial to acknowledge the combination of interests when it comes to thinking about the role of cities in cultural diplomacy. Thus, in the case of the present focus, one needs to consider the balancing act that the Moroccan state has had to perform in its international relations with three macro-regions—Europe, the Arab world and, increasingly, Africa—and how all this plays out at the micro-scale of the city festival.

It is also important to carefully think through the idea of urban agency in the specific Moroccan context. If this were simply seen to be concentrated in the hands of a mayor or a local administration, then one would conclude that little such agency exists in Morocco. While decentralization has occurred under Mohammed VI, progress has been hampered by the persistence of centralized control over decision-making and resource allocation, and for the time being, city governments have a very limited say in either urban development or cultural policy (Bouabid and Iraki 2015; Houdret and Harnisch 2019). Rather, we need to pay attention to the extent to which different local actors—including cultural entrepreneurs and activists—engage and contend with national institutional representatives and corporate sponsors in shaping the organization of city festivals and the transmission of their cultural messages to different audiences. A central argument here is that, in Morocco, the nation state is indeed very present in cultural diplomatic efforts at the urban level and that the characteristics and connections of a city are a fundamental conduit for communicating a range of discourses about a changing nation such as modernization, organizational capacity, religious tolerance, cultural diversity and Morocco’s rapprochement with the African continent. At the same time, the particularities of the urban context and local claims and counter-claims to cultural meaning shape the opportunities and constraints through which festivals are able to work towards cultural diplomatic goals. Hence, the paper ultimately aims both to assess how festivals in Moroccan cities are harnessed for their ‘cultural diplomatic capital’ and how these same spaces are implicated in the reconfiguration of ‘state-city relationships’ (Therborn 2011, p. 275).

### Urban Festivals and Cultural Diplomacy

There is a copious literature, especially in the field of geography, that critically examines the economic, political and symbolic relationship between festivals and urban development, including their role in place marketing strategies and growth-oriented entrepreneurial agendas as well as their implication in the gentrification of urban areas (Quinn 2005; Waitt 2008; Van Aalst and van Melik 2012). The contemporary growth of urban festivals across the world is seen to reflect ‘changes in the logic of capitalism’ (Waitt 2008, p. 516), insofar as they serve to enhance a city’s competitiveness and attractiveness, even if this paradoxically involves the duplication of events and reinforces social and cultural hierarchies within and between cities. A more sympathetic analysis by cultural sociologists instead considers festivals in the West as instances of the cultural public sphere and sociability that can work to foster cultural and political debate and community building (Giorgi et al. 2011).

In contrast, there has been little sustained reflection on the urban festival as a platform for cultural diplomacy, although see Hugoson (2015) for a discussion of the European Capitals of Culture programme as constituting a ‘transnational diplomatic arena’. Thinking about the direct and indirect diplomatic dimensions of festivals requires us to draw on both the ‘economic/developmental’ and ‘cultural sociological’ perspectives: on the one hand, the festival can showcase the organizational capacity in a city and thus be part and parcel of a place marketing strategy; on the other, it can provide a space in which cultural debate and relations are enacted. Indeed, a measure of a festival’s success as a vehicle for cultural diplomacy is the extent to which it is able to combine a range of goals. At the same time, the co-existence of potentially dissonant ambitions—for example, lavish spectacle and intercultural dialogue—suggests that the accumulated diplomatic capital of festivals is an inherently contradictory and precarious one.

This last point alerts us to the tension between interpretative and analytical approaches that underscores a lot of research on festivals and the pitfalls of relying on certain (economic) indicators that leaves little space for nuanced analysis (Sassatelli 2011). For example, how far are we be able to gauge the diplomatic intent of a festival when this is not always explicitly stated? Furthermore, cultural diplomacy is something that is ultimately immeasurable in terms of ‘success’ as stated intentions rarely tally with the often unexpected outcomes. Nevertheless, and taking on board the caveat of overinterpretation, it is precisely the multidimensional complexity of the festival that renders it an enticing space to grapple with the various facets of cultural diplomacy at the urban scale and the conflicts that might exist between them. This latter aspect is particularly pronounced in cities outside the West where festivals are often caught between the jostling for a position in a global cultural marketplace traditionally skewed by Western modes of consumption, and their ‘use value’ of facilitating cultural engagement and social cohesion in societies marked by a dearth of cultural facilities, under-funded services and deep disparities in wealth.

### Moroccan Festivals During an Era of Democratisation and Shifting International Relations

Morocco’s annual jamborees of the post-independence era—such as the long-running National Festival of Popular Arts in Marrakech—celebrated regional crafts and arts and were geared to strengthening the bond between the population and the monarchy as well as instilling an embryonic sense of national identity (Boum 2012). During the last two decades, cultural festivals in urban settings have rocketed. (See Table 1 for a list of predominantly annual festivals in Morocco’s ten largest cities during 2019, over 80% of which were established after 2000.) Many of these festivals have adopted a decidedly global, secular and contemporary direction and while they celebrate Morocco’s place in the world, they appear, on the surface at least, to be less overtly instrumental to nation building than in the past.

**Table 1:** Festivals in Morocco’s ten largest cities in 2019

Month	Festival	Length (days)	City	Year estd.
January	Laughing Africa [Comedy festival; also included dates in Abidjan, Bamako and Dakar]	3	Marrakech, Rabat, Casablanca	2017
February	International Youth Cinema Festival	5	Meknes	2011
March	National Film Festival International Festival of Contemporary Dance International Animated Film Festival International Festival of Theatre and Cultures Handifilm Festival [Disabilities-themed cinema]	9 16 6 11 3	Tangier Marrakech Meknes Casablanca Rabat	1982 2004 2004 2006 2007
April	Issni N’Ourgh International Amazigh Film Festival Caftan 2019 [Traditional costume/fashion festival] International Video Art Festival Jidar – Toiles de Rue [Street art festival] International University Festival of Cinematic Arts National Education Film Festival	5 1 6 7 3 5	Agadir Casablanca Casablanca Rabat Agadir Fès	2006 1996 1994 2015 2017 2002
May	RAMADAN			
June	Festival of North African Cinema Marrakech du Rire [Comedy festival] International Arab Film Festival World Festival of Sacred Music International Documentary Film Festival Mawazine [International popular music] Morocco Hip Hop Festival	5 5 8 9 5 9 5	Oujda Casablanca Meknes Fès Agadir Rabat/Salé Agadir	2012 2001 2019 1994 2008 2001 2018
July	Maroc Hikayate [Storytellers festival] National Festival of Popular Arts Jazzablanca Sbagha Bagha Street Art Festival Timitar Festival [Amazigh music] International Festival of Traditional Folklore International Festival of Amazigh Culture International Raï Festival Just for Laughs Festival Nights of Laughter Festival	13 5 6 17 4 4 3 5 4 2	Rabat Marrakech Casablanca Casablanca Agadir Agadir Fès Oujda Agadir Oujda	2004 1960 2005 2014 2004 2008 2004 2007 2017 2015
August	African Games* Volubilis International Festival of World Music	13 4	Rabat Meknes	1965 1999
September	World Salsa Congress International Festival of Urban Culture Oasis Festival [Electronic music] L’Boulevard Festival [Rock/Electro/Hip hop) Tanjazz International Women’s Film Festival Jazz au Chellah Rabat Art Biennale [24 September – 18 December]	7 14 3 10 8 6 5 86	Marrakech Meknes Marrakech Casablanca Tangier Salé Rabat Rabat	2014 2004 2015 1999 2000 2004 1996 2019
October	Mediterranean Short Film Festival Astronomy Festival International Theatre Festival Arab Film Festival Le Concert pour le Tolérance Festival of Sufi Culture National Amateur Film Festival Samaâ Festival of Sufi Encounters and Music	6 7 6 8 1 8 4 8	Tangier Marrakech Oujda Casablanca Agadir Fès Oujda Marrakech	2002 2000 2008 2018 2006 2008 2015 2006
November	International Contemporary Dance Festival International Author Film Festival Visa for Music - African Middle-East Music Meeting	14 7 4	Meknes Rabat Rabat	2019 1994 2013
December	International Film Festival Festival of Film and Migration Sun Festival [Youth arts and music] International Bird Festival International Travel and Adventure Film Festival Gharnati Music Festival	9 6 7 5 3 4	Marrakech Agadir Marrakech Kenitra Rabat Oujda	2001 2003 2008 2015 2016 1993

*The 12th African Games was the first to be hosted by Morocco, and the first time Morocco had participated in the quadrennial event since 1978. This sporting event has been included here to underline the renewed significance of Morocco’s relationship with Africa

The current ‘culture of festivalization’ (El Maarouf 2016) is both a product and a reflection of the myriad changes that have occurred in Moroccan politics and society. Under the helm of King Mohammed VI, who succeeded to the throne in 1999, the Moroccan state has embarked on a lengthy and carefully controlled programme of democratization, ostensibly guided by a commitment to human rights and greater social justice and marked by a desire to project the image of a modern, politically stable and culturally and economically dynamic nation in the global arena. This has involved a plethora of reforms, ranging from the involvement of civil society organizations in policy-making processes and the revision of family law to the official recognition of the nation’s non-Arab identities and the introduction of immigration policy. In part, these reforms respond to a groundswell demand for greater freedom and rights, but also a strategic manoeuvre by the Royal Palace to maintain domestic consensus, to court international favour and to stymie challenges to its power, in particular those posed by the rise of Islamism. In addition, a major switch on the foreign policy front has seen Morocco look beyond its traditional allies in Europe and the Gulf region to build closer economic, security and political ties with, among others, sub-Saharan Africa. This ‘African reconnection’, crowned by Morocco’s admission to the African Union in 2017 after an absence of more than 30 years, has been accompanied by a state-driven discourse about Morocco as an integral part of a culturally and economically dynamic continent (Abouzzohour and Tomé-Alonso 2019).

As an enthusiastic and extremely wealthy patron of the arts, Mohammed VI has frequently stressed the need to strengthen the place of culture in Moroccan public life (TelQuel 2013). The Moroccan state has likewise made a concerted push to rebrand Morocco as an international champion of cultural awareness and diversity. The increasing prominence assigned to culture over the last decade has had a decidedly urban character and has centred, in particular, on the country’s principal cities. A number of important factors are at play here. First, Morocco is not dominated by a single city but has historically possessed a polycentric urban network comprising a range of cities. Second, these cities have distinct identities and functions that readily translate into ‘urban cultural capital’ (Savage et al. 2018): Casablanca as the modern economic metropolis; Rabat as the seat of administrative and political power; Fès as the spiritual capital, Tangier as the Mediterranean gateway city; Marrakech as the global tourist and exotic heritage destination; and Agadir as the modern seaside resort and capital of Amazigh (Berber) culture. Third, since the era of the French protectorate, these cities have been central to Morocco’s international tourist industry, and have lately benefitted from the liberalization of the airline market with the European Union. Finally, flagship infrastructural projects in Rabat and Casablanca, including new museums, theatres and libraries, have been built in parallel to the redevelopment schemes and heritage restoration programmes that have occurred in all of the country’s major cities, underlining the increasingly urban-centric nature of Morocco’s economic development.

Against this backdrop, the urban festival format has played a key part in both enhancing Morocco’s international profile and expanding its cultural offer. From the perspective of their organizers and sponsors, festivals meet a series of immediate goals. They represent a significant tourist attraction, even for smaller urban centres such as Essaouira whose Gnawa music festival draws around 500,000 visitors to the coastal city each year, and are commonly seen to contribute to local economic development through the creation of jobs and spin-off activities. The raison d’être of many festivals is also aligned with broader political and symbolic targets. This has meant, first and foremost, transmitting messages to national and international audiences about a tolerant and culturally diverse Morocco that is at the same time open to the rest of the world. But it also includes cultivating ‘festival identities’ that reinforce a city’s existing reputation or provide it with a new vocation which, either way, can be conducive to place marketing. Hence, Casablanca’s various independent and alternative music festivals exalt its reputation as a dynamic, cosmopolitan and modern metropolis (Moreno Almedia 2017), while the world-renowned Festival of Sacred Music in Fès, premised on the values of international peace and dialogue, simultaneously reasserts the city’s status as the spiritual centre of the nation (Kapchan 2008; McGuiness 2012).

Urban festivals in Morocco, however, also face common dilemmas. A key task is how festivals conciliate their top-down approach (many are directed by individuals with close connections to the Royal Place and are financially supported by the King himself or else enjoy royal patronage that allows access to sponsorship and advertising) with the fact that the majority of the Moroccan population does not attend such events (Nouri and Sammouni 2016). The often ostentatious and smooth-working displays offered by the festivals contrast starkly with the country’s severely under-funded cultural sector, manifested by the paucity of public cultural facilities outside the central districts of major cities, and by the fact that in 2013, there were only 45 operating cinemas in the entire country, compared with 265 in the late 1970s (TelQuel 2013; for an overview of the evolution of Moroccan cultural policy, see Touzani 2016). Organizers of large free festivals in particular have had to fend off public criticisms of the co-optation of cultural expression and political dissent (Boum 2012), the squandering of state funds (El Maarouf 2016) and, specifically from the Islamist PJD party, accusations of immoral impropriety and material excess, which points to the tensions between secular and religious definitions of culture during the reign of Mohammed VI (Graiouid and Belghazi 2013). Already in 2008, Deborah Kapchan noted that festivals in Morocco were at the ‘center of moral, political, religious scrutiny’ (Kapchan 2008, p.471), and yet the number of new events continually rose thereafter, and this onward march would only meet its match during the 2020 lockdown when all festivals scheduled between March and September were abruptly cancelled.

The cultural diplomatic mission of urban festivals is thus framed by opportunities and constraints. To explore these dimensions further, I turn my attention to two festivals: the Marrakech International Film Festival and the Mawazine Rythmes du Monde music festival in Rabat. The former largely targets a global cultural elite and has sought to locate Marakkech and Morocco within international circuits of film consumption and production, while the latter is aimed at the general public and presents itself as the largest free music event in the world.

### The Marrakech International Film Festival: Local Sophistication and National Worldliness

The Festival International du Film de Marrakech (FIFM) was established in late 2001 on the orders of Mohammed VI with the aim of bringing international cinema to Morocco and boosting the cultural and tourist attractiveness of Marrakech (FIFM 2020a). It also set out to promote the surrounding region, in particular the studios in nearby Ouarzazate, as an open and safe setting for foreign film crews and—in the immediate aftermath of the 9/11 attacks—to reiterate Morocco’s commitment to the West and anti-terrorism. The first edition of FIFM was organized by the French film producer and personal friend of Mohammed VI, Daniel Toscan du Plantier, and was initially modelled on the mix of international stardom, sophistication and commerce that characterized the Cannes film festival. In 2002, the FIFM Foundation was established in order to provide an institutional framework for the festival, to allow for its evolution and to control the rights over its various activities. The Foundation’s board of directors have included members of the Makhzen, national government ministers, chairpersons of national companies such as Maroc Telecom and heads of regional government (although not the city’s mayor). As well as guaranteeing the festival with financial support, the Foundation has defined the FIFM’s central cultural messages, particularly through the public pronouncements of its president, the King’s brother and cinephile, Prince Moulay Rachid. The programming of FIFM, however, has been coordinated mainly by European festival professionals. For example, the artistic director appointed for the 2020 edition was Rémi Bonhomme, a French national and former manager of Cannes Critics’ Week who began his career as a cultural officer at the French Institute in Beirut.

Unlike other established festivals in the region, such as Carthage in Tunis and FESPACO in Ouagadougou which focus on, respectively, Arab and African cinema, FIFM is not a themed festival but has been conceived since the outset as a hybrid event that combines the red carpet trappings of international star-studded productions with an exposure to the ‘wider cultural and artistic experience of world cinema’ (FIFM 2020b). As well as vying with traditional heavyweight festivals in the Global North such as Cannes, Venice and Berlin, FIFM has carefully cultivated its reputation around the city of Marrakech and state-endorsed discourses about cultural diversity and dialogue. Hence, while its global ambitions are reflected in the ubiquitous use of English and French in both publicity and subtitles, the festival has introduced initiatives that enhance its claim to be a cultural crossroads, including annual tributes to cinematic traditions outside the West such as Egypt and Morocco itself, and the Atlas Workshop, launched in 2018, which is dedicated to supporting the regional film industry. It is this crafting of a cosmopolitan identity—as well as the generous hospitality lavished on its invited guests—that is cited by international jury members to be FIFM’s key allure, as illustrated by jury president James Gray’s press conference to Moroccan journalists in 2018 (Matin 2018).

The festival both harnesses and amplifies the pre-existing status of Marrakech as a glamorous and exotic destination, especially among Western visitors, and has widely been seen to offer an alternative experience to the tread-worn European festival circuit (Babana-Hampton 2011; Iordanova and Van de Peer 2014). FIFM and Marrakech have together produced a broad range of diplomatic resources. International film critics have commended FIFM’s sophisticated but at the same time laid-back celebration of cinema in a safe, tolerant and eminently accessible city (Malcolm 2003; Lumholdt 2007). Its rapid accumulation of prestige has worked to draw attention both to a fledgling national film industry and to official claims about Morocco as a regional bastion of freedom of cultural expression. FIFM’s official patrons, including Moulay Rachid himself, have continually drawn on stock phrases of the Mohammed VI era, such as fostering ‘an intercultural bridge between nations’ (FIFM 2020a) and celebrating Morocco’s ‘openness towards the world’ (Moulay Rachid 2013). In addition, the festival is presented as democratizing access to culture. For example, a giant screen is erected in the city’s main square Jemaa el-Fnaa during each festival projecting a selection of films for free to the general public. While this might be considered a cosmetic measure (considering the dearth of cinemas in the country), it is also a deliberate tactic that plays on the absence of such initiatives in other major—and socially exclusive—film festivals in the West.

The cultural diplomatic mission of FIFM is nevertheless constrained by a number of contradictions and public criticisms that point to the deep social and economic inequalities in both Marrakech and Morocco. The semblance of a ‘democratic’ festival has often been a source of contention, especially among local film makers and cinema goers who stress instances of censorship of Moroccan films, institutional controls over proceedings and the ostentatious expenditure and special treatment reserved for international stars (see, for example, the caustic reflection ‘Scorsese More at Home in Morocco than a Moroccan’ by the filmmaker Nadir Bouhmouch (2013), whose own film on the 20 February protests during the Arab Spring had been barred from Moroccan festivals). Over its lifespan, the festival has also been dogged by a conspicuous tension between a colonial legacy and postcolonial autonomy. Hence, while FIFM appears to intercept global cinema repertoires on ‘Moroccan’ terms and the organizing committee has usually included local experts within its ranks, to some observers and local film makers the event continues to feel like it is ‘programmed and run out of Paris’ (Iordanova and Van de Peer 2014, p. 53). Finally, the festival has spotlighted and, as a consequence, provided official countenance to the growing gentrification and westernization of Marrakech, particularly in the ancient medina in the city centre where the festival’s main venues are located and where, since the 1990s, thousands of properties have been acquired by European elites, many of which have since been converted into upmarket hotels and Riads that accommodate, among others, attendants of the International Film Festival (Escher and Petermann 2013).

### The Mawazine Festival in Rabat: Grandstanding a New African Capital

Mawazine Rythmes du Monde was established in 2001 and, from its inception, has been organized by the Maroc Cultures Foundation (MCF), a locally based non-profit organization focused on the ‘development and democratization of culture’ (Association Maroc Cultures 2019a). Since 2006, at the personal behest of Mohammed VI, MCF has been headed by the King’s personal secretary and Rabat-born businessman Mounir Majidi. Due to its huge dimensions, location in the nation’s capital and direct connections with the Palace, Mawazine, more than any other event, has been most directly associated with the cultural politics of festivals during the Mohammed VI era.

Originally envisaged as a celebration of ‘world music’ and targeted at a narrow public, Mawazine was rebranded after 2006 as an international popular music extravaganza for the masses. Besides maintaining its original commitment to showcasing non-Western acts especially from Africa, Mawazine broadened its remit to include big-name international stars as well as established Moroccan musicians and up-and-coming talent across a range of genres including rap and electronic dance music. Accompanying its basic brief to bring a range of music to a Moroccan and global audience, Mawazine’s organizers have sought to transmit a set of values. These are listed on the event’s official trilingual (Arabic, French and English) website as follows: ‘Sharing and Exchage: encourage interaction between artists and public and between artists’; ‘Tolerance and Openness: guarantee a large representation of different cultures’; ‘Diversity of Styles and Art: open up as widely as possible to all artistic expressions’; ‘Traditions and Modernity: combine traditional arts with most modern expressions’; ‘Quality and Authenticity: preserve cultural heritage’; and ‘Accessibility: enable each and everyone access free of charge to 90% of shows’ (Association Maroc Cultures 2019b).

According to MCF, Mawazine ‘has become a festival with strong civic dimension, that offers a real alternative in a country where the music industry is non-existent, where the market suffers from piracy and where budgets of the Ministry of Culture are limited’ (Association Maroc Cultures 2019c). As well as openly acknowledging the nation’s cultural deficiencies, this declaration captures a patent shift in cultural statecraft from the previously austere situation under Hassan II, who famously held private concerts inside the Royal Palace, towards a more ‘civic dimension’ whereby the practice and consumption of music is brought out into the urban public sphere. In 2019 Mawazine attracted 2.75 million spectators, which on paper makes it the largest music festival on the planet; an accolade that features prominently on the homepage of the event’s website and has prompted organizers to compare Mawazine with internationally-acclaimed events such as Glastonbury in the UK and Cochella in the USA.

Mawazine is also at the forefront of a drive to transform Rabat from its formerly drab status as a political and administrative centre into a global cultural hub. This process has involved the creation of new cultural institutions, such as the Mohammed VI Museum of Modern and Contemporary Art and the Zaha Hadid-designed Grand Théâtre, as well as globally oriented events such as the Rabat Art Biennale. At the same time, Mawazine reinforces Rabat’s position as capital of the nation, illustrated by the ubiquity of Moroccan symbols and the fact that many musicians, including international artists, drape themselves in the national flag at the end of their shows. Mawazine contributes to Rabat’s reconfiguration as both a world capital and a global metropolis and its simultaneous promotion as a bridge between Africa and the West. The geography of the festival is indicative. The international music stage where western acts perform is located in the upper-middle-class neighbourhood of Souiss, while the Moroccan music stage is across the river in Salé, Rabat’s poorer and more populous twin city. This alone would appear, as some have suggested, to reproduce a global hierarchy of value (Moreno Almedia 2017). However, one also needs to bear in mind that the Africa stage is mounted along the refurbished river bank beneath Rabat’s historic centre, which, significantly, is the focal point of the city’s current redevelopment. Other festivals in Rabat have similarly reflected an ‘institutionalized pan-Africanism’ (El Guabli 2018) that seeks to demonstrate the Moroccan state’s active interest in the continent’s economic, religious and cultural affairs, such as the annual Visa for Music meeting, which acts as both a manifesto for the capital’s cultivation of a post-Arab identity and an advertisement for Morocco’s relaxed visa regime to encourage the mobility of African artists.

Mawazine has received favourable coverage in the international press. For example, in 2014, *The Guardian* published an article entitled ‘How Rabat’s Mawazine music festival is signalling progress for the city’ (Razavi 2014), which applauded the global mix of music, free access to concerts and regeneration projects in the city. At the national level, however, Mawazine has also been the most contested festival in Morocco, encountering public opposition from civil society groups, media outlets and Islamists. Criticism has been levelled at the massive expenditure required to stage Mawazine in a country (and city) riddled with deep social and economic problems, although MCF stresses that the festival is funded by private sponsorship and ‘variable revenue’ such as advertising and no longer receives public subsidies (Association Maroc Cultures 2019c). It is notable that these subsidies were eliminated during the year of the Arab Spring, when pro-democracy protests in Rabat singled out Mawazine as a symbol of Royal extravagance and, according to public rumours, led the King to briefly consider cancelling the 2011 edition (TelQuel 2012). Furthermore, the idea that Mawazine embodied the democratization of music has been regularly censured and lampooned in online social media and has been supplanted with representations of the event as a modern-day, Moroccan version of bread and circuses (El Maarouf 2016). Numerous musical performers, especially in the rap community, have also disputed the claim that the festival functions as a springboard for new Moroccan talent because the palace-endorsed individual responsible for the line-up has persistently invited the same famous artists who guarantee large crowds and tend to indulge Moroccan patriotism (Moreno Almedia 2017).

On a separate front, Mawazine has been regularly berated for its western and secular excesses by Islamists, including members of the moderate Justice and Development Party (PJD) that has been in national government since 2011. This was exemplified most infamously in the PJD’s official protest against Elton John’s invitation to the festival’s 2010 edition on account of the singer’s homosexuality (Graiouid and Belghazi 2013). The incident became a global media story and gave Mounir Majidi, as the festival’s artistic director, the opportunity to reiterate the event’s underlying message: the star’s appearance was ‘necessary to promote diversity and cultural tolerance in Morocco’ (BBC News 2010). The Islamists’ accusations of *fujur* (debauchery) were not simply outmanoeuvred but were also contained by the state’s own self-restraint (the said concert went ahead but references to the artist’s sexuality were subsequently downplayed). Moreover, the fact that a ‘Boycott Mawazine’ campaign organized by Islamists in 2018 failed to make any headway (Guerraoui 2018)—in contrast to a successful parallel boycott of products of state-favoured companies—indicates the fragmented and incompatible positions that have characterized opposition to the festival and has ultimately provided further fuel to official claims about Morocco’s adherence to the freedom of expression.

## Conclusion

This paper has considered the relationship between cities, festivals and cultural diplomacy in the context of Morocco where the central state has assumed a commanding role in codifying and legitimating cultural practices. It has highlighted the different ways in which the urban scale enables the creation and transmission of selected cultural messages to different national and international audiences, and it alerts us to ways in which city-level cultural diplomacy operates beyond the spaces, such as transnational municipal networks, that are conventionally identified in the literature. At the same time, I have stressed the importance of adopting a pluralistic perspective regarding the notion of ‘urban agency’, one that considers the assemblage of different public and private actors but also the conflicts that are involved in shaping cultural diplomatic practices. This is crucial in order to avoid equating urban agency solely with the mayor and local administration (which in Morocco have had a very limited say in cultural matters), and to ward off the temptation of reifying the city itself as a unitary actor. Such an approach also requires us to pay particular heed to the rescaling of the state; in other words, how state power—the Monarchy and Mahkzen in the case of Morocco—harnesses and works through the local level as a means to legitimate its power and to redefine the significance of different cities vis-a-vis shifting ideas about the nation *and* the reconfiguration of international relations. Indeed, as shown, Morocco’s major cities have acquired distinct ‘festival identities’, while events, such as the International Film Festival in Marrakech and Mawazine in Rabat, have reinforced the particular strategic roles that these cities have started play on the international stage.

It could be argued in line with Koenraad Bogaert’s analysis of urban redevelopment projects in Rabat and Casablanca, that festivals are also ‘instrumental in burnishing the image of Moroccan exceptionalism characterized by good governance, social inclusion, participatory development and authoritarian moderation’ (Bogaert 2018b). Bogaert argues that the creation of special state agencies for managing urban redevelopment at the local level represents ‘more than just a shift in state power from the national level to the city. The global elites [i.e. investors] involved in local politics do not so much weaken state authority as become active players within these new state agencies. In this sense, authoritarianism in Morocco was not undermined by economic liberalization; it was re-institutionalized and rescaled, with power shared with new global actors’ (ibid.).

In a similar way, the urban festival has played its own part in the rescaling of cultural politics that has occurred during the reign of Mohammed VI, with the additional function of communicating these changes to national and international publics. As such, festivals can be understood as particular spaces of cultural power, where state actors and their private and civil society allies re-exert influence through the ‘soft’ guise of festive discourses and practices.

Despite the palace’s sponsorship and direct or proxy management of major urban festivals, the cultural diplomatic missions of such events are neither pre-determined nor smooth, but contradictory, contested and exposed to the unintended consequences that accompany the creation of spaces of cultural representation and exchange. Just as the Moroccan state has learnt to ‘talk the talk’ of western (cultural) diplomacy, so cultural stakeholders and activists have negotiated the limits and possibilities of the festival format, and have challenged their intended messages, such as democratization, intercultural dialogue and freedom of expression.

Ultimately, as US-based anthropologist Aomar Boum has argued, ‘for the Moroccan state, […] the festivalization of arts and culture has been a key method of managing dissent, blunting the force of social movements and sanding the political edges off of new forms of cultural expression – all while polishing an image of Morocco as liberal and fun-loving for outside consumption’ (Boum 2012, p. 25). At the same time, however, it is precisely the tensions between the secular and the religious and between cultural elites and the urban poor that often get revealed during festivals that makes these events particularly valuable for understanding the relationship between cities and the practice of cultural diplomacy in the specific non-Western context of Morocco.
